# Five reasons COVID-19 is less severe in younger age groups

**DOI:** 10.1093/emph/eoaa050

**Published:** 2020-12-26

**Authors:** Paul W Turke

**Affiliations:** Turke & Thomashow Pediatrics, 7444 Dexter-Ann Arbor Rd, Dexter, MI, US

## Abstract

The severity of COVID-19 is age-related, with the advantage going to younger age groups. Five reasons are presented. The first two are well-known, are being actively researched by the broader medical community, and therefore are discussed only briefly here. The third, fourth, and fifth reasons derive from evolutionary life history theory, and potentially fill gaps in current understanding of why and how young and old age groups respond differently to infection with SARS-CoV-2. Age of onset of generalized somatic aging, and the timing of its progression, are identified as important causes of these disparities, as are specific antagonistic pleiotropic tradeoffs in immune system function.

## Introduction

Infection with the novel coronavirus, SARS-CoV-2, runs the gamut from asymptomatic to lethal. It is well-established, however, that the risk of severe disease (including death) is markedly higher in older adult age groups than it is among children and young adults [1]. Five reasons for these age-related disparities will be identified and discussed.

## Five advantages for the young

*First*, obesity, hypertension, type 2 diabetes, kidney failure, cancer, and heart disease all become more prevalent as populations age, and mounting evidence indicates that each can contribute to poor clinical outcome in individuals infected with SARS-Co-V-2 [2]. Younger populations have fewer of these comorbidities, and therefore have a reduced likelihood of developing severe disease. Infected children with comorbidities nevertheless should be less negatively impacted by them than older adults because they lack some of the factors, below, which likely act synergistically with preexisting conditions.

*Second*, young children, especially those attending daycare centers, catch far more colds than other age group, and included among the responsible pathogens are 4 coronaviruses related to SARS-CoV-2 [3,4]. Past infections with coronaviruses are known to generate B and T cell memory, and it seems likely (though currently unproven) that this confers a degree of protective cross-immunity to SARS-CoV-2 [5,6,7]. Thus, if protective cross immunity indeed exists, but wanes over time, children are likely to have more of it than adults, particularly elderly adults.

Additional studies to determine the extent of protective cross immunity would be welcome, but even if protection is considerable, it would be unable to account for the exponential increase in severity and mortality in old age.

*Third*, aging (i.e., internal physiological deterioration) is absent in children and minimal in young adults. It accelerates, however, in later years, resulting in a generalized, progressive decline in somatic function [8,9,10]. Decreased resilience to all manner of insults, including infection, inevitably follows.

Immune system aging is a major contributor to the risk of developing severe COVID-19, and will be discussed separately below, but generalized age-related decline in all organ systems also can contribute to a poor outcome. Consider this potentially risky combination: aging is known to impair both cardiac function and damage-repair capabilities [11]; and SARS-CoV-2 is known to cause myocardial damage that at times severely compromises cardiac function [12,13]. It therefore seems likely that COVID-19 related cardiac insults such as cardiomyopathy and ischemia generally will be of greater consequence to the elderly than to the young.

It also seems likely that there will be, in some instances, or perhaps even most instances, a positive synergy between baseline age-related dysfunction, the comorbidities listed above, and infection with SARS-CoV-2. Although unsubstantiated at present, the overzealous inflammation (e.g., cytokine storm) and coagulation that can occur with COVID-19, most often in the elderly (see below), would seem to be particularly strong candidates for multiplying coexisting cardiovascular (and other) risks. The shape of the COVID-19 mortality curve directly implicates aging as a factor [14]; and its late exponential rise is consistent with the proposition that positive synergies contribute.

*Fourth*, a prodigiously active fetal and neonatal thymus rapidly builds a broad array of naïve T cells with unique receptors poised to recognize and respond to tens of millions of different antigens. This breadth of potential recognition and response narrows, however, over the life course, as responding naïve T cells convert to memory T cells, and as the thymus involutes and becomes unable to replenish the supply [15,16].

It seems clear, nonetheless, that having the ability to generate memory to past infections by converting naïve T cells to memory T cells is adaptive overall, since it has evolved and been maintained across diverse vertebrate taxa. What, though, are the specific benefits and costs? On the benefit side, memory T cells respond more quickly and vigorously to previously encountered antigen, and, importantly, this often generates a relatively near-term payoff. This is because the majority of conversions to memory are, perforce, due to encounters with pathogens that are common in the environment—endemic and seasonal pathogens that likely will be re-encountered again and again, often without much delay. On the cost side, the ineluctable narrowing of the naïve T cell repertoire, slowly, but progressively decreases the ability to recognize and respond to pathogens that have not yet been encountered, which, perforce, are increasingly likely to be rare pathogens, or even yet to emerge pathogens, as days without encounters accumulate. Thus, memory—the most important feature of the adaptive immune system—is intrinsically antagonistically pleiotropic [17,18].

There is as of now no direct evidence confirming (or contesting) the prediction that a broad naïve T cell repertoire confers an advantage in recognizing and countering, specifically, SARS-Co-V-2. There is, however, evidence of the importance of T cell breadth in dealing with novelty. For example, newly emergent cancer cells are novel threats, and work in mice completed almost 3 decades ago indicates that T cell conversion to memory status eventually leaves a depleted pool of naïve T cells, in turn leaving elderly mice especially vulnerable to newly arisen cancer cells [19]. More recently, and in this case in humans, age-related decline in T cell repertoire breadth has been shown to impair immunity to influenza [20]. Fully characterizing how this decline in breadth might affect recognition and response to SARS-CoV-2 is clearly an area of research that needs to be prioritized, if age-related disparities in COVID-19 severity are to be better understood [15,16].

*Fifth*, theory and evidence establish that natural selection is most potent early in lifetimes [8,9,10]. Theory and evidence also establish that many genes, both in humans and other organisms, have age-specific effects [8,9,10]. Together, these two facts predict that when novel selection pressures arise, new mutations that produce an adaptive response in young bodies will spread more rapidly and assuredly than if the same response is expressed only in older bodies.

The basis for this prediction is straightforward. To illustrate, consider two different mutations that produce an identical advantageous effect, but at different times of life, due to age-related variation in, say, growth hormone (GH) level, which is one of many components of the somatic environment known to regulate gene expression [21]. If the first mutation happens to express its effect only when GH is at a high level, which is during childhood, when the force of selection is at a maximum, whereas the second mutation expresses its effect downstream, only after GH has reached a very low level, which is late in adulthood, when selection has weakened, the first will spread and become fixed in the population more rapidly and more assuredly than the second. As proof of principle, a recent experiment has elegantly supported this central tenet of life history theory by showing that fruit flies exposed to a novel diet evolve relatively quickly to thrive on it—while in their youth, but not in old age [22].

There of course has not been sufficient time for humans of any age to adapt specifically to SARS-CoV-2. There has been, however, roughly 10-12,000 years of selection pressure on the human immune system to effectively respond to diseases that only became common when our ancestors began to live in permanent settlements and sustain themselves by domesticating plants and animals.  These have been referred to as 'crowd diseases' [23].

As for human coronaviruses, the 4 mentioned earlier (which cause relatively mild seasonal colds), probably crossed into humans from bats sometime after we became farmers, and by some accounts caused pandemics of their own [24]. The selection pressures that resulted can be expected to have changed our immune responses to coronaviruses [14]; and in light of the life history theory considerations discussed immediately above, it seems straightforward to predict that mutations that confer an advantage in dealing effectively with human coronaviruses in general, including (potentially) SARS-Co-V-2, will have spread more rapidly and assuredly if the advantage is confined to young age-groups, rather than old age groups. How long it will take older adults to catch up, if they indeed are behind, is an open question.

If selection has been operating as just described, we should be able to identify specific, recently evolved adaptations that give children and young adults an advantage during the ongoing COVID-19 pandemic. Among the possibilities are modifications in the timing and intensity of immunological responses mediated by interferons, including those recently highlighted in an evolutionary mismatch model that compares bats and humans [14]. Another is improved control, during youth, of inflammatory responses mediated by cytokines (e.g., IL2, IL7, and TNF$\alpha$) and bradykinins [4,14,15,16,25,26,27]. And yet another is reduced expression in children of the ACE2 receptors that are entryways for SARS-Co-V-2 [4,15,16,25,26].

SARS-CoV-2 is not the only coronavirus that uses the ACE2 receptor to enter human cells. Others are SARS-Co-V and NL63. The former emerged in 2002, too recently to have contributed significantly to evolutionary changes in our immune systems. Coronavirus NL63, on the other hand, is among the 4 coronaviruses mentioned earlier that cause seasonal colds; it has a worldwide distribution, and phylogenetic analyses indicate it emerged from its animal host (probably a bat) to infect humans about 1000 years ago [28]. Thus, the length of time since it jumped to humans, and its subsequent wide-spread distribution, together suggest that it may have relevance as a selective pressure that has altered our interaction with coronaviruses, perhaps by reducing ACE2 expression in children, or by modifying our inflammatory responses.

Of course, the foregoing adaptations might be appropriately considered explanations in their own right for why youth confers an advantage in managing SARS-CoV-2, but they each also might be appropriately considered proximate explanations, or mechanistic explanations, with the ultimate reason for their evolution being the hypothesis outlined above. And while this 'ultimate-level' hypothesis has not been tested, searching for selection hotspots in regions of the human genome known or suspected to involve immune system development and regulation could be a potential avenue for doing so. Such data could reveal new treatment targets and as of yet undiscovered mechanisms that help children and young adults more effectively manage crowd diseases in general, and coronaviruses specifically.

On the latter front, children as a group manage the inflammatory response to SARS-CoV-2 better than the elderly during the early stages of infection, but collateral damage from hyperinflammation, or mistargeted inflammation, occurs nevertheless in a very small proportion of children, roughly a month after initial infection. This is referred to as multisystem inflammatory syndrome in children (MIS-C), and it would be interesting and potentially useful to determine whether there are underlying genetic differences between the vast majority of children who avoid MIS-C and those who do not.

**Discussion**

Of the five proposed advantages that younger age-groups have in avoiding severe outcomes when infected with SARS-CoV-2, the first two have been in the spotlight throughout much of the COVID-19 pandemic, and are relatively well-supported. In contrast, the third, fourth and fifth reasons embody hypothetical explanations that are to varying degrees new, but which accordingly are only minimally supported by the available evidence. I suggest that they have credibility, nevertheless, by virtue having been straightforwardly derived from well-established tenets of evolutionary life history theory; and this, in my view, is the most compelling reason why they deserve additional vetting.

There of course almost certainly are additional explanations for age-related disparities in COVID-19 severity. One of these, also derived from life history theory (and thus also an ultimate-level hypothesis), may prove to be very significant. It rests on the idea that pre-pubertal children, because they have not yet begun to expend much effort on reproduction, are best able to focus on somatic maintenance [14,29].

Before concluding it is important to consider how the proposed 'five advantages' of youth can be evaluated, both for validity and significance. An initial approach would be to determine whether or not they produce their predicted age-specific proximate effect during infection with SARS-CoV-2. For instance, it should be feasible to establish, as has been done for influenza (see discussion above, and reference 20), whether breadth of the naïve T cell repertoire gives an advantage in recognizing and eliminating SARS-CoV-2, and concomitantly whether the narrowing of the repertoire that occurs with age creates deficits in recognition and clearance. As another example, it should be feasible to determine whether generalized somatic aging, age-related comorbidities, and infection with SARS-CoV-2 act synergistically to multiply risks for the elderly, as predicted.

In addition to the foregoing suggested tests, which focus exclusively on SARS-Co-V-2, a broader approach might include additional pathogens, as well as nonhuman hosts. For example, an experiment testing the validity of specifically the 'fifth advantage' could be done in fruit flies by exposing them to a novel pathogen over dozens of generations (using a design similar to the experiment briefly discussed earlier in which flies were presented with novel diets; see reference 22). The prediction, here, is that young flies would adapt more rapidly to the introduced pathogen than old flies. However, there is a caveat: unlike the referenced novel diet experiment, a potential complicating factor bearing on the predicted outcome for a newly introduced pathogen is that a coevolutionary arms-race might ensue, resulting in the introduced pathogen evolving counter adaptations, thus potentially making recently evolved host adaptations, and their corresponding adaptive effects, more difficult to recognize.

A variety of comparative tests also should be contemplated. Since the 'five advantages' are expected to be applicable to many infectious diseases, a logical prediction would be that younger age-groups will have advantages in managing the majority of them. However, morphing this prediction into one predicting less morbidity and mortality for different categories of youth (neonates, infants, toddlers, adolescents, and young adults) is not straightforward. There are many confounding variables that potentially weaken or even reverse this prediction that must be taken-into-account.

Foremost, is immunological memory to specific pathogens, which is absent, or minimal, in the very young. As has been argued, acquired memory is an extraordinarily valuable trait to have at the ready when encountering common, recurrent pathogens; so much so that it potentially can outweigh the advantages of youth proposed here and elsewhere (e.g., see references 14 and 29). Seasonal influenza illustrates the point. Although there is variation from year-to-year, very young children, who likely are experiencing influenza for the first time, tend to fare particularly poorly, both in terms of hospitalizations and deaths; older children and young adults, in contrast, usually have had prior experience with influenza viruses, and on that account do far better as a group [30].

Whereas the presence or absence of acquired memory is a major determinant of outcome in a multitude of diseases, there are also many variables that are disease-specific, and age-specific. These can significantly impact outcomes, too, but are harder to recognize because they are linked to specific host or pathogen attributes. To illustrate, consider infection with rotavirus and *Corynebacterium diphtheriae*, respectively.

Rotavirus is a leading cause of death throughout the less developed world, because it often causes prolonged diarrhea and vomiting, which together pose a risk for severe dehydration and shock, especially during infancy [31]. Infants are disadvantaged in preventing these dangerous outcomes, compared to older children and adults, because they generally will not yet have acquired memory to this pathogen, but also because they have a relatively high metabolic rate, a relatively large surface area compared to volume, and the fluids available to them for rehydration often are limited. Thus, although the advantages of youth that have been proposed are likely to confer a degree of help in combating rotavirus, they are sometimes insufficient to overcome the intrinsic increased risk of dehydration and death that follow from the variables just listed.

Diphtheria is another important crowd disease of relatively recent origin, and it is known to be especially severe in children under the age of 5 [32]. Here, the primary disadvantage is morphological: young children have a relatively small diameter airway—one that is easily occluded by the pharyngeal membrane that is pathognomonic for this disease. The all-too-common result is death by asphyxiation.

## Conclusion

Despite the many complexities that bear on infectious disease outcomes, it seems reasonable to suggest that greater focus on the contour of the force of natural selection over the course of the human lifespan will turn out to be broadly relevant to understanding age-related differences in host-pathogen interactions and outcomes, not just for SARS-CoV-2, but also for other crowd diseases of recent origin. A large-scale epidemiological study of the age-specific mortality rates that occurred when various crowd diseases were *first introduced into virgin populations* (a stipulation that removes the possibility of older groups having the advantage of acquired memory) would be an important first step in evaluating the validity and significance of the 'five advantages' presented above, as well as the more general claim that evolutionary life history will shed considerable light on host-pathogen interactions.

## Acknowledgment

I thank the editor of EMPH, two anonymous reviewers, and especially Bernie Crespi for sharing several important ideas, and for adding touches of clarity to my arguments. I also thank Laura Betzig, Margaret Nesse, Randy Nesse, Bev Strassman, and Claudius Vincenz for early encouragement that led me to write down my thoughts.
